# Hypoxia extends lifespan but does not alter telomere length or oxidative stress in a solitary bee (*Megachile rotundata*)

**DOI:** 10.1242/jeb.250500

**Published:** 2025-06-18

**Authors:** Andre Szejner-Sigal, Britt J. Heidinger, Aurelia C. Kucera, Jeffrey D. Kittilson, Alex S. Torson, Joseph P. Rinehart, George D. Yocum, Julia H. Bowsher, Kendra J. Greenlee

**Affiliations:** ^1^ Biological Sciences Department, North Dakota State University, Fargo, ND 58108-6050, USA; ^2^ USDA-ARS Edward T. Schafer Agricultural Research Center, Biosciences Research Laboratory, Fargo, ND 58102, USA

**Keywords:** Diapause, Reactive oxygen species, Cellular senescence, Quiescence, Aging, Overwintering

## Abstract

Stress can influence lifespan in both positive and negative ways, depending on exposure intensity and duration. However, mechanisms driving positive stress effects on lifespan remain poorly understood. Prolonged hypoxia extends the lifespan of overwintering prepupal *Megachile rotundata*. Here, we explored telomere length and reduced oxidative stress as potential mechanisms of this extended lifespan. We hypothesized high antioxidant capacity under hypoxia reduces oxidative damage and telomere loss. We exposed prepupae to 10%, 21% or 24% oxygen for up to 9 months and measured monthly survival, telomere length, antioxidant capacity and lipid peroxidation across treatment duration for prepupae and adults. After 9 months of exposure, survival was highest in hypoxia and lowest in hyperoxia. Telomere length did not differ among oxygen treatments but increased in adults compared with prepupae. Total antioxidant capacity and lipid peroxidation showed no significant differences among oxygen treatments, suggesting compensatory responses to maintain baseline oxidative levels.

## Teaser

**Summary:** Prolonged hypoxia in *Megachile rotundata* prepupae increases survival but has no effect on telomere length and oxidative stress, suggesting compensatory responses to maintain oxidative homeostasis.

## INTRODUCTION

Lifespan can be affected by stress both positively (Abdelrahman et al., 2014; Fontana et al., 2010; Muradian, 2013; Rascón and Harrison, 2010) and negatively (Aubert and Lansdorp, 2008; Finch and Kirkwood, 2000), depending on the type, timing, duration and intensity of the exposure. Abdelrahman et al. (2014) found that prolonged hypoxia almost doubled the lifespan of post-diapause quiescent prepupae of the leaf-cutter bee, *Megachile rotundata.* This observation provides an opportunity to explore the mechanisms by which hypoxia protects against cellular senescence. In this study, we assessed how prolonged hypoxia affects telomere length and oxidative stress, which are common measures of cellular aging.

Diapause and post-diapause quiescence are physiological states associated with dormancy during adverse conditions. Diapause is regulated by both genetic and environmental factors, while quiescence is driven solely by environmental conditions (Koštál, 2006). Both states are adaptations often linked to high-stress tolerance when compared with direct-developing individuals (King and Macrae, 2015) and are traits of interest for industry because of individuals' long-term viability and low maintenance requirements (e.g. *Trichogramma brassicae* for agricultural pest control, and *M. rotundata* for pollination). Abdelrahman et al. (2014) examined how post-diapause quiescent prepupae responded to different oxygen conditions. They showed that bees kept under prolonged hypoxia (10% oxygen) had significantly higher survival rates after nearly 2 years in quiescence compared with those in normoxia (21% oxygen) or hyperoxia (24% oxygen). Prolonged hypoxia did not affect flight metabolic rate or adult body size. The sublethal effects of hypoxia were a decrease in adult feeding rate and a reduction in adult female lifespan. The current study aimed to build upon that foundation to identify potential mechanisms, including telomere length and oxidative stress, that may drive the lifespan extension observed in post-diapause quiescent prepupae under prolonged hypoxia.

For aerobic organisms, hypoxia directly interferes with ATP production by disrupting the mitochondrial respiratory chain and can cause the release of reactive oxygen species (ROS) (Murphy, 2009; Solaini et al., 2010). ROS are highly reactive molecules that can damage biomolecules, including lipids, proteins and DNA, and can result in reduced lifespan in many organisms, including some insects (Agarwal and Sohal, 1994; De Loof, 2011; Monaghan et al., 2009; reviewed by Shields et al., 2021). However, ROS can also play regulatory roles in various essential cell processes such as oxygen sensing (Chen et al., 2021; Hamanaka and Chandel, 2009; Sena and Chandel, 2012). To offset the negative effects of increased ROS, cells upregulate the production of antioxidants (enzymes and small molecules) that prevent oxidative damage. The imbalance between ROS and antioxidants is referred to as oxidative stress (Monaghan et al., 2009; Sies et al., 2017).

In addition to its deleterious effects on macromolecules, exposure to oxidative stress has also been shown to accelerate telomere loss both *in vitro* and *in vivo* (Epel et al., 2004; Herborn et al., 2014; Sohal et al., 1993; von Zglinicki, 2002). Telomeres are highly conserved, repetitive DNA sequences at the ends of linear chromosomes that play an important role in cellular lifespan (Monaghan, 2010; Monaghan and Haussmann, 2006). As cells continuously replicate, telomeres gradually shorten to the point where cells stop dividing and secrete inflammatory compounds (Zhang et al., 2016), making telomere length a useful biological aging marker. Telomere loss is thought to contribute to declines in tissue function with age and to overall organismal aging, with evidence suggesting that telomere length during early life can predict lifespan (Heidinger et al., 2012; Vaiserman and Krasnienkov, 2021). Telomere length has been shown to increase with age in alfalfa leafcutting bees (Grula et al., 2024), but it is unclear how hypoxia may affect this relationship.

Although hypoxia can induce a release of ROS (Clanton, 2007; Guzy et al., 2005), mild hypoxia has been found to reduce oxidative damage to proteins in *Drosophila melanogaster* and increase maximum lifespan (Rascón and Harrison, 2010). However, most studies that show benefits to low oxygen involve short-term exposures and very low levels of oxygen (<5%) (Berry and Lopez-Martinez, 2020; Campbell and Lopez-Martinez, 2022). In this study, we tested the hypothesis that prolonged hypoxia triggers a strong response that counteracts increased ROS production and decreases telomere shortening, ultimately leading to increased lifespan in post-diapause quiescent prepupae of *M. rotundata.* Specifically, we predicted that (1) exposure to prolonged hypoxia would decrease the rate of telomere loss compared with normoxia and hyperoxia, resulting in longer telomeres for bees exposed to hypoxia, and (2) the oxidative response (indicated by an increase in antioxidants, a decrease in oxidative damage, or both) would be highest in hypoxia, and return to baseline levels upon completion of development in normoxia. To test these hypotheses, we measured prepupae and adult telomere length after prolonged hypoxia, normoxia and hyperoxia and quantified total antioxidant capacity and lipid peroxidation levels across oxygen treatments and exposure duration.

## MATERIALS AND METHODS

### Rearing of overwintering prepupae

Prepupal *Megachile rotundata* (Fabricius 1787) were purchased from JWM Leafcutter, Inc. (Nampa, ID, USA) in 2015, and stored according to the industry standard protocol (6°C in constant darkness). Prepupae were kept in their brood cells and maintained in constant darkness at 6°C for 9 months. At that time, following Abdelrahman et al. (2014), brood cells were placed in a fluctuating thermal regime (6°C, with a daily pulse to 20°C) prior to the start of the experiment to induce prolonged prepupal lifespan (Abdelrahman et al., 2014, Rinehart et al., 2011, 2013; Bennett et al., 2013).

### Oxygen treatments

Post-diapause quiescent prepupae (*n*=3000) were haphazardly assigned to one of three treatment groups: hypoxia (10% O_2_), hyperoxia (24% O_2_) and normoxia (21% O_2_) (Dataset 1: Survival). Brood cells were placed on one of two mesh trays (*n*=500 for each tray) and placed into a modular hypoxia chamber (Billups-Rothenberg, Del Mar, CA, USA). There were two chambers for each gas treatment for a total of 6 chambers (*n*=1000 for each gas treatment). Twice a week, chambers were flushed with the assigned gas for 5 min with a flow rate of 20 l min^−1^ (10% and 24% O_2_) or opened to room air for 5 min (21% O_2_). Hypoxic and hyperoxic gases were premixed and certified (Praxair Specialty Gases, Hillsdale, IL, USA) prior to the experiment with their respective percentage of oxygen and the balance nitrogen. For each treatment, we collected monthly subsamples of 75 individuals from month 16 to month 24 of quiescence, excised them from brood cells, and snap froze them in liquid nitrogen for further analysis. To determine survival, we also collected monthly subsamples of 120 individuals from each treatment and placed each brood cell in an individual well of 24-well culture plates. We transferred these individuals to 29°C with constant darkness and 75% humidity to resume development through to adult emergence. Survival was quantified as successful adult emergence from the brood cell after 2 months at 29°C. We flash-froze a sample of 75 adults in liquid nitrogen within 24 h of emergence for further analysis. All frozen bees were kept at −80°C until used for molecular analysis.

### Telomere length

We measured relative telomere length (*T*/*S* ratio) in prepupae and adults for individuals after 1 and 7 months of exposure (i.e. 16 and 22 months in quiescence), using protocols modified from Cawthon (2002) and Grula et al. (2024) using quantitative PCR (qPCR) (Dataset 2: Telomere length). Briefly, we extracted DNA from whole bodies of prepupae and adults using a Nucelospin Insect DNA Extraction Kit (Macherey-Nagel, Allentown, PA, USA) to measure relative telomere length. To quantify DNA concentration, we used a NanoDrop 1000 spectrophotometer (ThermoFisher Scientific), and samples with *A*_260_/*A*_280_ ratios below 1.8 were not used. For qPCR, we used a Mx3000P qPCR system (Agilent, Santa Clara, CA, USA) to quantify relative telomere length using the *T*/*S* ratio, which compares the telomere signal (*T*) with a single control gene (*S*). For *M. rotundata*, we used the single-copy gene glyceraldehyde-3-phosphate dehydrogenase (GAPDH) (BioProject accession no.: PRJNA66515), as the control gene and the conserved (TTAGG)n telomere sequence, and primers and concentrations of each as used by Grula et al. (2024).

The telomere and GAPDH reactions were run using the protocol from Grula et al. (2024). Briefly, plates containing GAPDH primers were run for one cycle for 10 min at 95°C, 35 cycles for 20 s at 95°C, 39 s at 59°C and 30 s at 72°C, and one cycle for dissociation. For telomere plates, we ran one cycle for 10 min at 95°C, 35 cycles for 20 s at 95°C, 39 s at 58°C and 30 s at 72°C, and one cycle for dissociation. All reactions used 20 ng of DNA in a final volume of 25 μl containing 12.5 µl of SYBR green Master Mix (PerfeCTa SYBR Green SuperMix Low ROX, Quantabio Beverly, MA, USA), 0.25 µl forward and reverse primers, 6 µl water and 6 µl of DNA sample. We used a pool of five individuals to make a 5-point standard curve (40, 20, 10, 5, 2.5 ng) used across all plates. On each plate, we used water as a negative control and a non-treatment control. All samples were randomized across plates and run in duplicate, and standard curve data in triplicate. The average efficiency for telomere and GAPDH plates was 103.7% and 95.3%, respectively.

### Total antioxidant capacity

We assessed total antioxidant capacity (TAC, enzymatic and non-enzymatic) using the Cayman Chemical Antioxidant Assay Kit (Ann Arbor, MI, USA) following protocols used by Torson et al. (2019) (Dataset 3: TAC). Each bee sample was homogenized in 400 µl PBS (pH 7.4) with a mixture of 0.5 mm and 1.0 mm zirconium oxide beads for 3 min in a Bullet Blender Blue (Next Advance, Averill Park, NY, USA). The homogenized samples were centrifuged at 10,000 ***g*** for 15 min at 4°C and then 350 µl of the homogenate was decanted from the microcentrifuge tube and aliquoted for storage at −80°C until needed for TAC, lipid peroxidation and protein quantification assays. For TAC, samples were diluted 1:5 in assay buffer. We followed the manufacturer's protocol for the rest of the assay and absorbance was measured at 405 nm in duplicate using a microplate reader (BioTek Synergy H1, Agilent, VT, USA). Results are expressed as mmol l^−1^ Trolox equivalents mg^−1^ protein. We standardized to total protein content using a modified Bradford assay (Bio-Rad, Berkeley, CA, USA), with absorbance read at 595 nm against a 0.05–0.5 mg ml^−1^ bovine serum albumin (BSA) standard curve. Samples were diluted 1:40 in PBS prior to protein measurements.

### Lipid peroxidation

We measured thiobarbituric acid reactive substances (TBARS), a marker of oxidative lipid damage, using a TBARS (TCA method) Assay Kit (Cayman Chemical, Ann Arbor, MI, USA), and following protocols used by Torson et al. (2019) (Dataset 4: TBARS). A 10 µl aliquot of each of the homogenized samples (described in the previous section) was diluted 1:5 with RIPA buffer (250 mmol l^−1^ Tris-HCl, pH7.6, containing 750 mmol l^−1^ NaCl, 5% NP-40, 2.5% sodium deoxycholate and 0.5% SDS) to ensure TBARS concentrations fell within the standard curve. The RIPA buffer was treated with Roche cOmplete™ protease inhibitor cocktail tablets (Roche, Indianapolis, IN, USA). We ran reactions in duplicate, and TBARS concentrations were calculated from a 0–50 nmol ml^−1^ malondialdehyde (MDA) standard curve with absorbance read at 530 nm. We determined protein content using a modified Bradford assay (Bio-Rad), with absorbance read at 595 nm against a 0.05–0.5 mg ml^−1^ BSA standard curve, and then used this to standardize TBARS as μmol l^−1^ MDA mg^−1^ protein.

### Statistical analysis

All statistical analyses were performed in R v.4.4.0 (http://www.R-project.org/), and all data are presented as means±s.e.m. For the survival analysis, we fitted a generalized linear model (GLM) using a binomial distribution (emerged=1, not emerged=0) and a logit link function. The model included oxygen treatment, months in quiescence and their interaction to assess variation in survival across experimental conditions. For the molecular analysis, we fitted linear models with developmental stage, oxygen treatment, months in quiescence and their interactions as fixed effects. For telomere length analysis, we fitted linear mixed models using the *lme4* package, with the dependent variable as natural log-transformed telomere length and included developmental stage, oxygen treatment, month sampled and their interactions as fixed effects. We included plate ID as a random effect. We simplified models by dropping non-significant terms from the final models (Crawley, 2007; Sokal and Rohlf, 2003), and model improvement was tracked through the Akaike information criterion (AIC). When ΔAIC<2, the simplest model was selected due to parsimony. For models with significant interaction terms, we used pairwise comparison from the *emmeans* package with Bonferroni multiple-group corrections.

## RESULTS AND DISCUSSION

### Impact of prolonged hypoxia/hyperoxia on telomere length

**Fig. 1.**
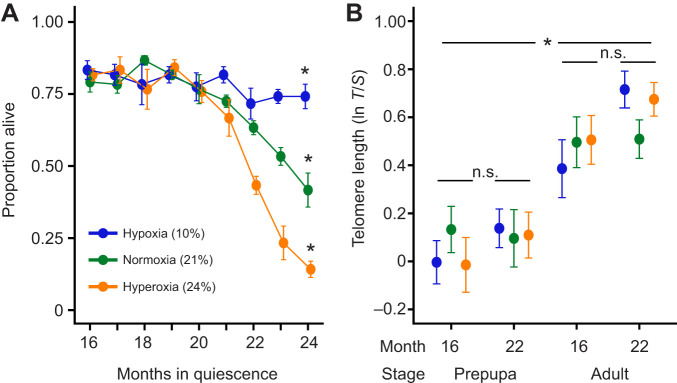
**Survival and telomere length of *Megachile rotundata* exposed to different oxygen conditions during post-diapause quiescence.** (A) Survival of bees from prepupae to adulthood (*n*=120 per treatment per month). OxygenxMonths in quiescence, for 10–21% *z*=−4.057, for 10–24% *z*=−8.668. (B) Telomere length (telomere signal *T*/single control gene *S*) for prepupal and adult stage of individuals quiescent for 16 and 22 months across oxygen treatments (*n*=19–26 per treatment per month). Stage, *T*-value=9.927. Treatments started at month 16, with prolonged exposure to hypoxia, normoxia or hyperoxia (10%, 21% or 24% oxygen, respectively). Data are means±s.e.m. and asterisks indicate significance (*P<0.01).

Bee prepupae under prolonged hypoxia had lower mortality and survived longer compared with those under normoxia and hyperoxia, which rapidly declined after month 19 of quiescence (Fig. 1A; oxygen×months in quiescence, *P*<0.01 for all comparisons; Table S1). These results replicate those found by Abdelrahman et al. (2014), where there was a strong negative association between prepupal survival and oxygen concentration. Insects undergoing diapause and post-diapause quiescence have high stress tolerance, as they decrease their aerobic metabolism and upregulate heat-shock proteins (Hahn and Denlinger, 2011; King and Macrae, 2015). Exposure to prolonged hypoxia allowed overwintering prepupae to extend their survival past 23 months, outliving most individuals in normoxia and hyperoxia (Abdelrahman et al., 2014).

Oxygen levels and treatment duration (exposed individuals at 16 and 22 months in quiescence) did not impact telomere length (Fig. 1B; 21% O_2_ *P*=0.58, 24% O_2_ *P*=0.79, months in quiescence *P*=0.18; Tables S2–S4). The absence of a relationship between telomere length and treatment was true for both prepupae and adults. Interestingly, adults had longer telomeres than prepupae (Fig. 1B; *P*<0.001). Telomere length usually declines with age across many taxa (Louzon et al., 2019), which means that longer telomeres in adults is a counter-intuitive result. However, this pattern was also found by Grula et al., (2024), who demonstrated that *M. rotundata* have longer telomeres as adults than post-diapause quiescent prepupae. Our observation of longer telomeres in adults confirms this pattern, and suggests that resuming development post-dormancy or processes during metamorphosis could result in adults with tissues that are cellularly younger as indicated by telomere length. Telomere length can be restored by the enzyme telomerase. In honey bees, telomerase activity is higher in adult queens than in workers, which also differ in lifespan by several orders of magnitude (Korandová and Frydrychová, 2016). In vertebrates, telomerase is down-regulated in somatic tissues leading to cellular senescence, and increased telomerase is associated with cell proliferation and observed in most human cancers (Ahmed and Tollefsbol, 2001; Blackburn, 2005). Hypoxia up-regulates telomerase in cancer cell lines (Guan et al., 2012; Yatabe et al., 2004), but the connection between hypoxia and telomerase in free-living organisms remains undetermined. Our results show no effect of oxygen on telomere length, but we encourage further studies on the oxygen sensitivity of telomerase in *M. rotundata*. If telomere dynamics follow cellular aging as adult bees senesce, restoring telomere length would likely be adaptive. Most somatic tissues do not grow in adult insects, and bee fitness is closely linked to flight performance, one of the most oxygen-demanding performances in the animal kingdom (Suarez, 2000). Telomere dynamics in other holometabolous and hemimetabolous insects could reveal novel patterns of cellular aging under varying life history contexts.

### Impact of prolonged hypoxia/hyperoxia on oxidative response

**Fig. 2.**
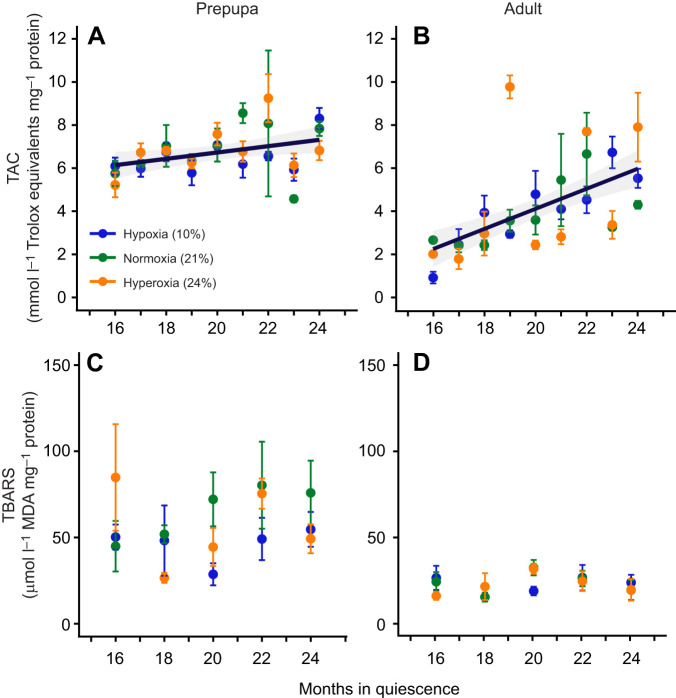
**Oxidative responses of *M. rotundata* exposed to different oxygen conditions during post-diapause quiescence.** Total antioxidant capacity (TAC; top) and lipid peroxidation (TBARS; bottom) for prepupae (A,C) and adults (B,D) after prolonged exposure to hypoxia, normoxia or hyperoxia (10%, 21% or 24% oxygen, respectively). Data are means±s.e.m., *n*=27 per treatment per stage for TAC and *n*=15 per treatment per stage for TBARS. Regression lines and 95% confidence interval (shaded region) show significant effects (*P*<0.01).

TAC during extended overwintering did not vary between oxygen treatments (21% O_2_ *P*=0.88, 24% O_2_ *P*=0.35; Tables S5 and S6) but was significantly affected by developmental stage and months in quiescence, with adults having a steeper increase in TAC the longer they remained in quiescence compared with prepupae (Fig. 2A,B; stage×month in quiescence, *P*=0.004; Tables S5 and S6). Lipid peroxidation did not differ between oxygen treatments or months in quiescence (21% O_2_ *P*=0.14, 24% O_2_ *P*=0.45, months in quiescence *P*=0.42; Tables S5 and S6), but prepupae had higher levels of lipid peroxidation compared with adults (Fig. 2C,D; stage, *P*<0.001; Tables S5 and S6). The absence of differences between treatments suggests a compensatory response to maintain ROS levels under prolonged hypoxia and hyperoxia at levels similar to those found in normoxic conditions, at least for lipid maintenance and TAC. This partially supports our hypothesis that hypoxia induces an oxidative response to counteract oxidative stress. Our results also support past findings that extended overwintering in *M. rotundata* in normoxia had no effect on antioxidant levels or oxidative damage (Torson et al., 2019). This suggests that oxidative levels (e.g. the combination of antioxidant and ROS levels) are tightly controlled during bee development, as they are maintained at baseline levels even under prolonged hypoxic and hyperoxic conditions. ROS levels play multiple regulatory roles in the cell, including oxygen sensing (Hamanaka and Chandel, 2009), and a recent study shows ROS levels can also regulate metabolic cycles during pupal diapause (Chen et al., 2021). Contrary to our prediction, the oxidative response under hypoxia was similar to that under hyperoxia, suggesting that oxidative stress alone does not explain the differences in prepupal lifespan. However, this study assessed only two oxidative markers, when oxidative stress involves hundreds of redox reactions occurring in a complex environment. For example, in honey bees, vitellogenin can act as an antioxidant and is strongly associated with increased lifespan in queen honeybees (Havukainen et al., 2013; Kodrik et al., 2023; Seehuus et al., 2006). Interestingly, extended-overwintering treatment in *M. rotundata* drives an upregulation of vitellogenin (Torson et al., 2019), but its role in prolonged hypoxia and its effect on lifespan remain unexplored.

Abdelrahman et al. (2014) showed that, upon resuming development after prolonged hypoxia, *M. rotundata* adults exhibited sublethal effects of hypoxia, including lower feeding rates and shorter female adult lifespans. Interestingly, in standard developmental conditions, prepupal energy stores primarily drive adult mass at emergence (Grula et al., 2021; Helm et al., 2017) and larger females live longer (Szejner-Sigal et al., 2025). However, body size and mass were not affected by prolonged hypoxia, even when prepupae lived for several months more (Abdelrahman et al., 2014). The uncoupling of energy use and lifespan in a seasonal context violates the general assumption that energy stores decline with longer periods of dormancy (Roberts et al., 2023). Our results also show that prolonged hypoxia does not induce disruption to oxidative status and development. Hypoxia can induce cell arrest (Harrison et al., 2006), which may further decrease metabolic demands, which are already low during quiescence. An increase in anaerobic metabolites would be associated with a switch in metabolic fuels during hypoxia, and not in normoxia and hyperoxia. We encourage future research to explore metabolic fuel dynamics during extended periods of dormancy that seem to come at no energetic cost, as these types of studies remain scarce.

When combining our results on telomere length and oxidative stress, we conclude that lipid peroxidation and total antioxidant capacity do not explain the increase in prepupal lifespan under prolonged hypoxia. This study adds to the growing evidence that oxidative stress alone does not solely explain lifespan extensions (Pithan et al., 2024; Shields et al., 2021; Viña et al., 2013). We found a compensatory response under hypoxia and hyperoxia that maintained oxidative levels similar to those under normoxic conditions, at least for total antioxidant capacity and lipid peroxidation. We highlight that the current understanding on telomere dynamics in holometabolous insects, and other animals with complete metamorphosis, remains widely understudied, while challenging general principles regarding the link between telomeres and biological aging.

## Supplementary Material

10.1242/jexbio.250500_sup1 Supplementary information

Dataset 1. Survival. Data of adult *Megachile rotundata* emergence when exposed to different oxygen conditions during post-diapause quiescence

Dataset 2. TS. Data of prepupa and adult *Megachile rotundata* telomere length when exposed to different oxygen conditions during post-diapause quiescence.

Dataset 3. TAC. Data of prepupa and adult *Megachile rotundata* total antioxidant capacity (TAC) when exposed to different oxygen conditions during post-diapause quiescence.

Dataset 4. TBARS. Data of prepupa and adult *Megachile rotundata* lipid oxidative stress (TBARS) when exposed to different oxygen conditions during post-diapause quiescence.
