# Supplementary material for: Hypoxia extends lifespan but does not alter telomere length or oxidative stress in a solitary bee (Megachile rotundata)
Source: J Exp Biol. 2025 Jun 18;228(12):jeb250500. doi: 10.1242/jeb.250500 (PMC12211587; doi:10.1242/jeb.250500)
Supplement: Supplementary information [file jexbio-228-250500-s1.pdf]

Table S1. Generalized linear model results of survival, oxygen treatments, and months in quiescence for Megachile rotundata bees.

**Model:** Survival ~ Oxygen * Months in quiescence				
**Effect**	**Estimates**	**S.E**	**z-val**	**P-val**
(Intercept)	2.510	0.569	4.406	<0.001
Oxygen 21%	2.791	0.802	3.480	<0.001
Oxygen 24%	6.345	0.842	7.531	<0.001
Months in quiescence	-0.066	0.027	-2.401	0.016
Oxygen 21%:months	-0.158	0.038	-4.057	<0.001
Oxygen 24%:months	-0.356	0.041	-8.668	<0.001

Table S2. Linear mixed model comparison and selection of telomere length, oxygen treatment, months in quiescence, and stage using AIC criteria and parsimony.

Model	AIC	logLik	Df	P-val	dAIC
**tsln ~ stage + (1 | plate)**	**331.1**	**-161.55**			**0**
tsln ~ stage + ox + (1 | plate)	334.7	-161.35	2	0.817	3.6
tsln ~ stage + ox + month + (1 | plate)	334.8	-160.42	1	0.171	3.7
tsln ~ stage + ox * month + (1 | plate)	338.5	-160.27	2	0.862	7.4
tsln ~ stage * ox * month + (1 | plate)	346.1	-159.05	5	0.945	15.0

Table S3. Linear mixed model results of relative telomere length, oxygen treatments, and months in quiescence for Megachile rotundata bees.

**Model:** Telomere length ~ Stage + Oxygen + Months in quiescence + (1 | plate ID)					
**Effect**	**Estimates**	**S.E (S.D.)**	**Df**	**T-val**	**P-val**
**Fixed effects** (marginal R^2 = 0.21, conditional R^2 =0.41)					
(Intercept)	0.058	0.088	270	0.654	0.513
Stage	0.485	0.049	270	9.877	<0.001
Oxygen 21%	-0.033	0.060	270	-0.553	0.581
Oxygen 24%	-0.016	0.061	270	-0.260	0.795
Months in quiescence 7	0.067	0.049	270	1.350	0.178
**Random effects**					
plate ID	0.054	(0.234)			
Residual	0.168	(0.41)			

Journal of Experimental Biology • Supplementary information

Table S4. Best fit linear mixed model of relative telomere length and developmental stage for Megachile rotundata bees.

**Model:** Telomere length ~ Stage + (1 | plate ID)					
**Effect**	**Estimates**	**S.E (S.D.)**	**Df**	**T-val**	**P-val**
**Fixed effects** (marginal R^2 = 0.21, conditional R^2 =0.41)					
(Intercept)	0.074	0.078	273	0.950	0.343
Stage	0.486	0.049	273	9.927	<0.001
**Random effects**					
plate	0.057	(0.238)			
Residual	0.167	(0.41)			

Table S5. Linear model comparison and selection of oxidative stress, oxygen treatments, months in quiescence, and stage using AIC criteria and parsimony.

Model	AIC	Sum of Squares	F	df	dAIC
Total Antioxidant Capacity					
Tac ~ stage * ox * month	670.26			13	2.66
Tac ~ stage + ox * month	670.55	-33.19	1.9679	8	2.96
Tac ~ stage * ox + month	670.55	-0.002		8	2.96
**Tac ~ stage + ox + month**	**667.59**	**-3.471**	**0.5144**	**6**	**0**
Lipid Peroxidation					
Tbars ~ stage * ox * month	811.802			13	5.1303
Tbars ~ stage + ox * month	808.4896	-2517.2	1.2033	8	1.8179
Tbars ~ stage * ox + month	807.7267	296.71		8	1.055
**Tbars ~ stage + ox + month**	**806.6717**	**-1159.34**	**1.3856**	**6**	**0**

Journal of Experimental Biology • Supplementary information

Table S6. Linear model results of oxidative stress, oxygen treatments, and months in quiescence for Megachile rotundata prepupa and adults.

Total Antioxidant Capacity (TAC)				
**Model:** TAC ~ Stage + Oxygen + Months in quiescence				
**Effect**	**Estimates**	**S.E**	**T-val**	**P-val**
(Intercept)	-2.182	1.168	-1.868	0.063
Stage - Prepupa	2.617	0.292	8.959	<0.001
Oxygen 21%	0.065	0.357	0.183	0.854
Oxygen 24%	0.431	0.357	1.207	0.229
Months in quiescence	0.306	0.056	5.414	<0.001
Lipid Peroxidation (TBARS)				
**Model:** TBARS ~ Stage + Oxygen + Months in quiescence				
**Effect**	**Estimates**	**S.E**	**T-val**	**P-val**
(Intercept)	1.792	15.943	0.112	0.910
Stage - Prepupa	33.08	4.339	7.624	<0.001
Oxygen 21%	10.07	5.314	1.895	0.061
Oxygen 24%	5.191	5.314	0.977	0.331
Months in quiescence	0.790	0.767	1.030	0.305

Dataset 1. Survival. Data of adult *Megachile rotundata* emergence when exposed to different oxygen conditions during post-diapause quiescence.

Available for download at
https://journals.biologists.com/jeb/article-lookup/doi/10.1242/jeb.250500#supplementary-data

Dataset 2. TS. Data of prepupa and adult *Megachile rotundata* telomere length when exposed to different oxygen conditions during post-diapause quiescence.

Available for download at
https://journals.biologists.com/jeb/article-lookup/doi/10.1242/jeb.250500#supplementary-data

Journal of Experimental Biology • Supplementary information

Dataset 3. TAC. Data of prepupa and adult *Megachile rotundata* total antioxidant capacity (TAC) when exposed to different oxygen conditions during post-diapause quiescence.

Available for download at
https://journals.biologists.com/jeb/article-lookup/doi/10.1242/jeb.250500#supplementary-data

Dataset 4. TBARS. Data of prepupa and adult *Megachile rotundata* lipid oxidative stress (TBARS) when exposed to different oxygen conditions during post-diapause quiescence.

Available for download at
https://journals.biologists.com/jeb/article-lookup/doi/10.1242/jeb.250500#supplementary-data
